# Dietary inflammatory index and the risk of gastric cancer in a Korean population

**DOI:** 10.18632/oncotarget.20008

**Published:** 2017-08-07

**Authors:** Sunghee Lee, Jeonghee Lee, Il Ju Choi, Young-Woo Kim, Keun Won Ryu, Young-Il Kim, Jin-Kyoung Oh, Binh Thang Tran, Jeongseon Kim

**Affiliations:** ^1^ Department of Cancer Biomedical Science, Graduate School of Cancer Science and Policy, National Cancer Center, Goyang, South Korea; ^2^ Center for Gastric Cancer, National Cancer Center, Goyang, South Korea; ^3^ Department of Cancer Control and Population Health, Graduate School of Cancer Science and Policy, National Cancer Center, Goyang, South Korea; ^4^ Department of Food and Nutrition, College of Health Science, Kangwon National University, Samcheok, South Korea

**Keywords:** inflammation, diet, gastric cancer, interaction, case-control study

## Abstract

We aimed to investigate the association with the Dietary Inflammatory Index (DII™) on the risk of gastric cancer and whether histological type modifies this association. From March 2011 to December 2014, 388 cases and 776 controls were enrolled at the National Cancer Center. Utilizing a food frequency questionnaire, thirty-five food components were used to score the DII. The tertile distribution of DII for controls was as follows: T1: <0.96, T2: 0.96-2.97, and T3: ≥2.97. To investigate the association between DII and the gastric cancer risk, multivariable logistic models were constructed. In subgroup analyses, histological types including intestinal and diffuse types were examined. As the DII increased, gastric cancer risk increased (*p-value* for trend =0.007). Participants in the highest DII tertile had a greater gastric cancer risk compared to those in the lowest tertile [Odds Ratio (OR) =1.63, 95% Confidence Interval (CI) 1.15-2.29]. Stratification by sex revealed that men who were in the highest DII tertile showed a greater risk of intestinal type (OR=2.03, 95% CI 1.09-3.77). Participants positive for *H. pylori* infection had higher risk of intestinal type (OR=2.16, 95% CI 1.21-3.87). In this case-control study, we found a significantly positive association with a pro-inflammatory diet on gastric cancer risk, after adjusting for covariates. Future studies are suggested to prospectively examine the effect of a pro-inflammatory diet on gastric cancer risk.

## INTRODUCTION

Gastric cancer is the fifth most prevalent cancer worldwide [1] and is the most common cancer among Korean men [2]. This high prevalence may be attributed to the high rate of *Helicobacter pylori (H. pylori)* positive infection, which occurs in approximately 54.4% [3] of Korean adults and to chronic inflammation from chronic gastritis, leading to mucosal dysplasia and gastric carcinogenesis. The inflammatory process increases the risk of the development of carcinogenesis [4, 5]. Chronic inflammation due to chronic atrophic gastritis and *H. pylori* infection plays an important role in developing gastric carcinogenesis [6]. Furthermore, a recent study indicated that the use of non-steroidal anti-inflammatory drugs (NSAIDs) such as low-dose aspirin as a preventive and treatment measure to inhibit inflammation reduced gastric cancer risk [7]. However, the use of the medications for individuals who are not at risk of heart problems or stroke may cause risky bleeding in the stomach or brain.[8] Thus, a dietary approach to reduce inflammation is safer and is recommended.

To reduce chronic inflammation related to the risk of cancer, many previous studies have reported pro- and anti-inflammatory effects of dietary approaches. A high intake of fruits and vegetables has been shown to reduce inflammation [9]. whereas a high intake of red and processed meats has been reported to increase inflammation [10-12]. Additionally, a ‘healthy dietary pattern’ has been indicated to reduce inflammation [13]. However, most previous studies assessed a single nutrient or a food group, which might not reflect the total effect of diet-related inflammation on cancer risk, as individuals consume combinations of nutrients. Accordingly, the Dietary Inflammatory Index (DII™) was developed to estimate the comprehensive diet-related inflammatory potential linked to a health outcome [14, 15]. The DII was designed to assess diet-related inflammation and was calculated from pro-inflammatory and anti-inflammatory dietary components based on studies of diverse populations worldwide [15]. The validity of the DII has been confirmed by comparing dietary data and high-sensitivity C-Reactive Protein (hs-CRP) in a large longitudinal study, which indicated that a high DII was associated with hs-CRP [16]. A study using follow-up data from the National Health and Nutrition Examination Survey III (NHANES III) in a general population reported significant associations with DII on all-cause, cardiovascular, and cancer-related mortality [17]. Studies have assessed the associations with DII on the risk of breast cancer [18], esophageal cancer [19, 20], colorectal cancer [21, 22], and prostate cancer [23]. Additionally, the Women’s Health Initiative (WHI), a longitudinal prospective cohort study, showed a higher risk of breast cancer mortality associated with a higher DII [24].

Only one previous study has examined the association between DII and gastric cancer risk; this study investigated, the relationship in a southern European population [25]. However, dietary patterns in southern Europe differ greatly from those in eastern Asia. The current study is unique in that it was conducted in a Korean population, which has a high reported prevalence of *H. pylori* infection--approximately 54.4% [3]. Furthermore, it is important to examine histologically different types of gastric cancer, as these types determine prognosis [26].

Therefore, the study objective of the current study was to investigate whether gastric cancer risk was associated with the DII and whether different histological types modified the association between the DII and gastric cancer risk among 1,164 study participants (388 cases and 776 controls). We hypothesized that individuals with a high DII have a greater gastric cancer risk and the individuals with a high DII as well as *H. pylori* positive infection have a greater gastric cancer risk. Additionally, a histological type modified the association with DII on gastric cancer risk in a Korean case-control study.

## RESULTS

### Descriptive characteristics

Table 1 presents the general descriptive characteristics among the study participants. Cases were diagnosed with gastric cancer, whereas the controls were not. The two groups showed no difference in the distribution of age and BMI. However, men with gastric cancer drank and smoked more than men without gastric cancer (both *p*<0.001). Additionally, the positive infection of *H. pylori* was significantly more prevalent among the cases than among the controls (*p*<0.001 for both men and women). By contrast, the frequency of regular exercise was lower in the cases than in the controls (*p*<0.001 for both men and women). The prevalence of a first-degree family history of gastric cancer differed significantly between cases and controls in men but not in women (*p=*0.001 for men and *p=*0.609 for women). Education was significantly associated with gastric cancer risk (*p*<0.001 for men and *p*=0.009 for women). In particular, the frequencies of men and women with greater than 12 years of education were lowest among the cases. Additionally, among men, the cases had significantly greater total energy intake compared to the controls (*p*<0.001). Among women, the cases had higher total energy intake than the controls, but the difference in total energy intake was not statistically significant (*p*=0.317). Among both men and women, DII scores were statistically higher in the cases than in the controls (*p*=0.017 for men and *p*=0.002 for women). Furthermore, the histological types of gastric cancer were assessed; the intestinal type was the most prevalent among men (53.28%), whereas the diffuse type was the most prevalent among women (66.67%).

**Table 1:** General characteristics of the study participants (n=1,164)

	Men (n=747)		*p*-Value	Women (n=417)		*p*-Value
	Case (n=249)	Control (n=498)		Case (n=139)	Control (n=278)	
Age, years	54.31±8.81	54.33±8.62	0.981	51.25±9.58	51.44±9.43	0.852
Body mass index, kg/m^2^	24.11±2.93	24.44±2.71	0.121	23.07±2.91	23.17±2.90	0.725
Smoking, pack-years	21.35±17.80	14.80±14.30	<0.001	0.59±2.91	0.47±2.63	0.654
Drinking, ethanol amount, g/day	28.87±44.26	16.6±26.67	<0.001	3.62±8.63	2.46±7.60	0.163
*H. pylori,* positive infection, n (%)	232 (93.17)	321 (64.46)	<0.001	124 (89.21)	157 (56.47)	<0.001
Regular exercise, n (%)	98 (39.36)	287 (57.63)	<0.001	37 (26.62)	143 (51.44)	<0.001
First-degree family history of gastric cancer, n (%)	61 (24.50)	71 (14.26)	0.001	20 (14.39)	35 (12.59)	0.609
Education						
≤ 9 years	83 (33.33)	68 (13.65)	<0.001	46 (33.09)	56 (20.14)	0.009
9 – 12 years	99 (39.76)	198 (39.76)		63 (45.32)	137 (49.28)	
≥ 12 years	67 (26.91)	232 (46.59)		30 (21.58)	85 (30.58)	
Dietary intake						
Total energy intake, kcal/day	2075.00±707.86	1775.27±559.89	<0.001	1691.86±511.56	1635.47±555.88	0.317
Dietary Inflammatory Index	2.53±2.20	2.13±2.13	0.017	2.02±2.27	1.31±2.21	0.002
Histological type						
Intestinal	122 (53.28)	-		24 (19.05)	-	
Diffuse	71 (31.00)	-		84 (66.67)	-	
Mixed	36 (15.72)	-		18 (14.29)	-	

Mean±S.D.

### Characteristics of the study participants according to DII tertiles

Table 2 presents the general characteristics of the study participants according to the DII tertiles. The DII increased as the average age of women decreased (*p*-value for trend =0.005). No trend was observed in BMI, drinking, prevalence of positive *H. pylori* infection, education, or frequency of first-degree family history of gastric cancer. However, as the DII increased, the frequency of regular exercise decreased (*p*-value for trend <0.001 for men, 0.005 for women). For the histological type of gastric cancer, men showed higher prevalence rates of the intestinal types throughout the tertiles, whereas women had higher frequencies of the diffuse types across tertiles.

**Table 2:** General characteristics of the study participants according to the tertile range of the Dietary Inflammatory Index (n=1,164)

	Men (n=747)			*p*-Value for trend	Women (n=417)			*p*-Value for trend
	Dietary Inflammatory Index tertile range				Dietary Inflammatory Index tertile range			
	T1	T2	T3		T1	T2	T3	
	n=204	n=228	n=315		n=150	n=147	n=120	
Age, years	54.51±8.24	55.08±8.71	53.65±8.90	0.198	52.78±8.72	51.48±9.52	49.49±10.05	0.005
Body mass index, kg/m^2^	24.48±2.83	24.44±2.69	24.16±2.82	0.179	23.40±2.87	23.14±3.17	22.81±2.56	0.100
Smoking, pack-years	14.74±14.63	17.35±16.41	18.17±16.09	0.019	0.20±1.73	0.56±3.41	0.82±2.78	0.061
Drinking, ethanol amount, g/day	23.48±42.67	21.38±33.47	18.38±27.48	0.089	2.70±6.81	2.64±6.87	3.28±10.27	0.576
*H. pylori*, positive infection, n (%)	148 (72.55)	164 (71.93)	241 (76.51)	0.271	100 (66.67)	92 (62.59)	89 (74.17)	0.230
Regular exercise, n (%)	120 (58.82)	128 (56.14)	137 (43.49)	<0.001	78 (52.00)	60 (40.82)	42 (35.00)	0.005
First-degree family history of gastric cancer, n (%)	29 (14.22)	38 (16.67)	65 (20.63)	0.155	18 (12.00)	16 (10.88)	21 (17.50)	0.245
Education								
≤ 9 years	36 (17.65)	42 (18.42)	73 (23.17)	0.107	37 (24.67)	35 (23.81)	30 (25.00)	0.968
9 – 12 years	75 (36.76)	89 (39.04)	133 (42.22)		70 (46.67)	74 (50.34)	56 (46.67)	
≥ 12 years	93 (45.59)	97 (42.54)	109 (34.60)		43 (28.67)	38 (25.85)	34 (28.33)	
Dietary intake								
Total energy intake, kcal/day	2014.73 ±500.72	1772.03 ±625.07	1859.46 ±688.65	0.018	1767.04 ±513.67	1514.42 ±555.12	1684.61 ±525.02	0.134
Dietary Inflammatory Index	-0.58±1.16	2.13±0.58	4.2±0.96	<0.001	-0.97±1.27	2.08±0.57	4.05±0.80	<0.001
Histological type								
Intestinal	23 (40.35)	37 (57.81)	62 (57.41)	0.073	6 (18.75)	4 (10.26)	14 (25.45)	0.481
Diffuse	21 (36.84)	15 (23.44)	35 (32.41)		21 (65.63)	29 (74.36)	34 (61.82)	
Mixed	13 (22.81)	12 (18.75)	11 (10.19)		5 (15.63)	6 (15.38)	7 (12.73)	

Mean±S.D; the tertile ranges of DII were categorized based on the distribution of the control group (T1: <0.96, T2: 0.96-2.97, T3: ≥2.97).

### The association between the DII and the risk of gastric cancer

Table 3 demonstrates the association between the DII and the risk of gastric cancer. After adjusting for potential confounding factors, gastric cancer risk increased as the DII increased (*p*-value for trend =0.007). Specifically, participants with DII scores in the highest tertile had 1.63 times gastric cancer risk compared to those with DII values in the lowest tertile [odds ratio (OR) = 1.63, 95% confidence interval (CI) 1.15-2.29].

**Table 3:** Associations between the Dietary Inflammatory Index and the risk of gastric cancer (n=1,164)

	Dietary Inflammatory Index			*p*-Value for trend	*p*-Value for interaction
	Tertile range				
	T1	T2	T3		
	n=354	n=375	n=435		
**Case/Control, n (%)** 388 (33.33%) /776 (66.67%)	96 (27.12) /258 (72.88)	116 (30.93) /259 (69.07)	176 (40.46) /259 (59.54)		
**Total** (n=1,164)					
Crude model	1.0 (ref)	1.20 (0.87, 1.66)	1.83 (1.35, 2.47)	<0.001	
Multivariable model	1.0 (ref)	1.41 (0.98, 2.03)	1.63 (1.15, 2.29)	0.007	
**Sex**					
**Men** (n=747)					
Crude model	1.0 (ref)	1.11 (0.74, 1.67)	1.42 (0.97, 2.07)	0.059	0.013
Multivariable model	1.0 (ref)	1.34 (0.83, 2.17)	1.31 (0.84, 2.05)	0.279	
**Women** (n=417)					
Crude model	1.0 (ref)	1.35 (0.81, 2.26)	3.06 (1.82, 5.14)	<0.001	
Multivariable model	1.0 (ref)	1.48 (0.83, 2.62)	2.98 (1.68, 5.30)	<0.001	
***H. pylori* infection**					
**Positive** (n=330)					
Crude model	1.0 (ref)	1.24 (0.87, 1.78)	1.65 (1.18, 2.32)	0.003	0.189
Multivariable model	1.0 (ref)	1.40 (0.95, 2.07)	1.50 (1.04, 2.16)	0.037	
**Negative** (n=834)					
Crude model	1.0 (ref)	1.53 (0.54, 4.36)	3.00 (1.12, 7.99)	0.021	
Multivariable model	1.0 (ref)	1.59 (0.52, 4.84)	2.93 (1.02, 8.43)	0.036	

Adjusted for total caloric intake, body mass index, education, smoking (pack-years), ethanol amount, physical activity, *H. pylori* infection, and first-degree family history of gastric cancer; The tertile ranges of DII were categorized based on the distribution of the control group (T1: <0.96, T2: 0.96-2.97, T3: ≥2.97).

When stratified by sex, compared to the lowest tertile of DII, the highest tertile was associated with an increased risk of gastric cancer in women but not men (OR=2.98, 95% CI 1.68-5.30 for women, OR=1.31, 95% CI 0.84-2.05 for men). Women also showed a significantly increasing trend for gastric cancer risk as DII increased (*p*-value for trend <0.001). Additionally, the variable ‘sex’ exhibited a significant interaction on the association with DII on gastric cancer risk (*p*-value for interaction=0.013).

When stratified by the presence of *H. pylori* infection, individuals with *H. pylori* positive and negative infection who had DII scores in the highest tertile demonstrated an increased risk of gastric cancer (OR=1.50, 95% CI 1.04-2.16 for positive infection, OR=2.93, 95% CI 1.02-8.43 for negative infection). Furthermore, *H. pylori* infection did not show a significant interaction (*p*-value for interaction=0.189).

### Subgroup analyses according to histological types of gastric cancer of the association between DII and the risk of gastric cancer stratified by sex and *H. pylori* infection

Table 4 presents the results of the subgroup analyses of participants with either intestinal or diffuse type gastric cancer. Overall, participants with a DII in the highest tertile showed 1.86 times gastric cancer risk compared to those with a DII in the lowest tertile after adjusting for potential confounding variables (OR=1.86 95% CI 1.28-2.70). Of participants with intestinal type gastric cancer, those in the highest DII tertile demonstrated 2.33 times the risk of gastric cancer after adjusting for confounding factors (OR=2.33, 95% CI 1.37-3.96), whereas among participants with the diffuse type, those in the highest DII tertile showed an increased risk but this increase was not statistically significant.

**Table 4:** Differences in Dietary Inflammatory Index (DII) according to histopathological types among the gastric cancer patients (n=301)

		All types (n=301)				Intestinal type (n=146)				Diffuse type (n=155)			
	No. of Controls, n (%)	No. of Cases, n (%)	Crude OR (95% CI)	Multi- variable OR (95% CI)	*p* for int.	No. of Cases, n (%)	Crude OR (95% CI)	Multi- variable OR (95% CI)	*p* for int.	No. of Cases, n (%)	Crude OR (95% CI)	Multi-variable OR (95% CI)	*p* for int.
**Total**	776 (72.05)	301 (27.95)				146 (48.50)				155 (51.50)			
T1	258 (33.25)	71 (23.59)	1.0 (ref)	1.0 (ref)		29 (19.86)	1.0 (ref)	1.0 (ref)		42 (27.10)	1.0 (ref)	1.0 (ref)	
T2	259 (33.38)	85 (28.24)	1.19 (0.83, 1.71)	1.42 (0.95, 2.12)		41 (28.08)	1.41 (0.85, 2.34)	1.86 (1.04, 3.32)		44 (28.39)	1.04 (0.66, 1.65)	1.14 (0.70, 1.86)	
T3	259 (33.38)	145 (48.17)	2.03 (1.46, 2.84)	1.86 (1.28, 2.70)		76 (52.05)	2.61 (1.65, 4.14)	2.33 (1.37 3.96)		69 (44.52)	1.64 (1.08, 2.49)	1.49 (0.95, 2.35)	
*p*-value for trend			<0.001	0.001			<0.001	0.002			0.017	0.077	
**Sex**													
**Men**	498 (72.07)	193 (27.93)			0.029	122 (83.56)			0.142	71 (45.81)			
T1	144 (28.92)	44 (22.80)	1.0 (ref)	1.0 (ref)		23 (18.85)	1.0 (ref)	1.0 (ref)		21 (29.58)	1.0 (ref)	1.0 (ref)	
T2	156 (31.33)	52 (26.94)	1.09 (0.69, 1.73)	1.40 (0.82, 2.38)		37 (30.33)	1.49 (0.84, 2.62)	2.19 (1.12, 4.28)		15 (21.13)	0.66 (0.33, 1.33)	0.76 (0.36, 1.60)	0.037
T3	198 (39.76)	97 (50.26)	1.60 (1.06, 2.43)	1.55 (0.95, 2.52)		62 (50.82)	1.96 (1.16, 3.31)	2.03 (1.09, 3.77)		35 (49.30)	1.21 (0.68, 2.17)	1.09 (0.57, 2.05)	
*p*-value for trend			0.017	0.086			0.011	0.046			0.399	0.724	
**Women**	278 (72.02)	108 (27.98)				24 (16.44)				84 (54.19)			
T1	114 (41.01)	27 (25.00)	1.0 (ref)	1.0 (ref)		6 (25.00)	1.0 (ref)	1.0 (ref)		21 (25.00)	1.0 (ref)	1.0 (ref)	
T2	103 (37.05)	33 (30.56)	1.35 (0.76, 2.40)	1.41 (0.75, 2.67)		4 (16.67)	0.74 (0.20, 2.69)	0.67 (0.16, 2.77)		29 (34.52)	1.53 (0.82, 2.85)	1.61 (0.81, 3.21)	
T3	61 (21.94)	48 (44.04)	3.32 (1.89, 5.84)	3.25 (1.75, 6.05)		14 (58.33)	4.36 (1.60, 11.92)	4.87 (1.47, 16.07)		34 (40.48)	3.03 (1.62, 5.66)	2.93 (1.47, 5.84)	
*p*-value for trend			<0.001	<0.001			0.003	0.008			0.001	0.002	
***H. pylori*** **infection**													
**Positive**	478 (63.65)	273 (36.35)			0.126	129 (88.36)			0.438	144 (92.90)			
T1	158 (33.05)	66 (24.18)	1.0 (ref)	1.0 (ref)		26 (20.16)	1.0 (ref)	1.0 (ref)		40 (27.78)	1.0 (ref)	1.0 (ref)	
T2	150 (31.38)	78 (28.57)	1.25 (0.84, 1.85)	1.45 (0.95, 2.23)		36 (27.91)	1.46 (0.84, 2.53)	1.85 (0.98, 3.51)		42 (29.17)	1.11 (0.68, 1.80)	1.19 (0.71, 1.98)	0.164
T3	170 (35.56)	129 (47.25)	1.82 (1.26, 2.62)	1.69 (1.13, 2.51)		67 (51.94)	2.40 (1.45, 3.96)	2.16 (1.21, 3.87)		62 (43.06)	1.44 (0.92, 2.27)	1.37 (0.85, 2.21)	
*p*-value for trend			0.001	0.012			<0.001	0.012			0.106	0.196	
**Negative**	298 (91.41)	28 (8.59)				17 (11.64)				11 (7.10)			
T1	100 (33.56)	5 (17.86)	1.0 (ref)	1.0 (ref)		3 (17.65)	1.0 (ref)	1.0 (ref)		2 (18.18)	1.0 (ref)	1.0 (ref)	
T2	109 (36.58)	7 (25.00)	1.28 (0.40, 4.18)	1.32 (0.38, 4.62)		5 (29.41)	1.53 (0.36, 6.56)	1.85 (0.39, 8.77)		2 (18.18)	0.92 (0.13, 6.64)	0.82 (0.10, 6.62)	
T3	89 (29.87)	16 (57.14)	3.60 (1.27, 10.21)	3.50 (1.14, 10.75)		9 (52.94)	3.37 (0.89, 12.84)	3.61 (0.86, 15.19)		7 (63.64)	3.93 (0.80, 19.42)	3.66 (0.66, 20.39)	
*p*-value for trend			0.009	0.016			0.057	0.065			0.057	0.076	

Adjusted for total caloric intake, body mass index, education, smoking (pack year), ethanol amount, physical activity, *H. pylori* infection, and first-degree family history of gastric cancer; The tertile ranges of DII were categorized based on the distribution of the control group (T1: <0.96, T2: 0.96-2.97, T3: ≥2.97); *p* for int.: *p*-value for interaction.

Regarding sex differences, men showed a significantly increased risk for the intestinal type in the highest tertile of DII compared to the lowest tertile of DII, after adjusting for confounding factors (OR=2.03, 95% CI 1.09-3.77). By contrast, women had a significantly increased risk of both intestinal and diffuse types associated with the highest DII tertile compared to the lowest DII tertile (OR=4.87, 95% CI 1.47-16.07 for the intestinal type, OR=2.93, 95% CI 1.47-5.84 for the diffuse type). Of the participants with *H. pylori* positive infection, those in the greatest tertile of DII had 2.16 times the risk of intestinal type gastric cancer than those in the lowest tertile (OR=2.16, 95% CI 1.21-3.87). No other significance according to histological types was observed. Overall, individuals with *H. pylori* negative infection exhibited stronger associations than *H. pylori* positive individuals (OR=3.50, 95% CI 1.14-10.75 for negative vs. OR=1.69, 95% CI 1.13-2.51 for positive).

## DISCUSSION

In this case-control study of 1,164 Korean adults, higher diet-related inflammation as assessed by the DII was strongly associated with a greater risk of gastric cancer, even after adjusting for potential confounding variables. In particular, when stratified by sex, a significant association with a higher DII on gastric cancer risk was observed among women. Additionally, regardless of *H. pylori* infection, individuals with DII scores in the highest tertile exhibited a greater gastric cancer risk. In the subgroup analyses based on histological type, men with higher DII values had a greater risk of the intestinal type, but women with higher DII scores had greater risks of both histological types. Additionally, when stratified by *H. pylori* infection, intestinal type was associated with greater DII scores only among individuals with *H. pylori* positive infection.

Our findings regarding the greater gastric cancer risk associated with higher DII scores are consistent with previous epidemiological studies, including those of colorectal cancer [21, 22], esophageal [19, 20], prostate cancer [23], and breast cancer [18]. Specifically, a recent case-control study in a Korean population demonstrated that colorectal cancer risk was associated with the highest tertile of DII compared to the lowest tertile (OR=2.16, 95% CI 1.71-2.73) [21]. Another case-control study on the risk of prostate cancer indicated a significantly increased risk associated with the highest quintile of DII compared to the lowest quintile of DII (OR=2.48, 95% CI 1.50-4.10) [23]. An increasing amount of evidence has demonstrated significant associations between DII and cancers in large populations. A 20-year longitudinal study of postmenopausal Swedish women found an increased risk of breast cancer with DII values in the highest quartile compared with the lowest [hazard ratio (HR)=1.22, 95% CI 1.01-1.46] [18]. Another longitudinal study, the Women’s Health Initiative, demonstrated in a 16 year follow-up of 122,788 postmenopausal women that DII increased the risk of breast cancer mortality (HR=1.33, 95% CI 1.01-1.76) [24]. Furthermore, in the most recent study by Shivappa et al., investigating the association with DII on gastric cancer risk in an Italian population, a higher DII increased the risk of gastric cancer [25]. Their study indicated that the highest quartile of DII had 2.35 times the risk of gastric cancer compared to the lowest quartile of DII (OR=2.35, 95% CI 1.32-4.20) [25], consistent with the current study (OR=1.63, 95% CI 1.15-2.29). Their study also utilized quartile categories of DII, whereas our current study used tertile ranges of DII. However, in contrast to our study, the previous authors did not assess *H. pylori* infection or histological types of gastric cancer. Moreover, our study participants resided in a setting in which gastric cancer is the most prevalent cancer among men and the second most prevalent cancer after thyroid cancer among women [2]. Additionally, the participants in the current study had a high prevalence rate of *H. pylori* positive infection and consumed different foods than the Italian population.

Unexpectedly, our current study indicated that individuals with *H. pylori* negative infection showed stronger associations between DII and the risk of gastric cancer than individuals with *H. pylori* positive infection. As pro-inflammatory or anti-inflammatory modulators, foods and dietary patterns play a role in increasing or decreasing the risk of gastric cancer. Previous studies related to the inflammatory potential of diets on gastric cancer have shown that dietary risk factors such as a high intake of salty foods or red meats increase the risk of gastric cancer [27, 28], whereas a high intake of fruits and vegetables [29], including dietary vitamin C intake [30], has been reported to reduce the risk of gastric cancer. In addition to these food components, dietary patterns have been shown to have an effect on the estimated risk of gastric cancer. A 10-year follow-up longitudinal study of 54,498 Japanese adults examined the effect of three dietary patterns on the risk of gastric cancer and found that the “traditional pattern”, which includes pickled vegetables, dried fish, salted gut, salted roe, miso soup, shellfish, fish, and rice, increased gastric cancer risk [relative risk (RR)=2.88, 95% CI 1.76-4.72 among men, RR=2.40, 95% CI 1.32-4.35 among women] [31]. Moreover, a meta-analysis of 8 studies reported that a “Western/unhealthy pattern” led to an increased risk of gastric cancer compared to a “prudent/healthy pattern” (OR=1.51, 95% CI 1.21-1.89) [32].

The etiology of cancers is strongly linked to chronic inflammation as a main contributor to the development of carcinoma [4, 5]. Gastric cancer can also develop via chronic inflammation resulting from chronic atrophic gastritis, intestinal metaplasia, or dysplasia [5, 33]. Thus, controlling levels of chronic inflammation in the stomach via dietary intake may mitigate subsequent steps leading to the development or progression of gastric carcinoma.

The findings of our subgroup analyses based on different histological types are consistent with previous reports [26]. Regarding sex differences, the intestinal type of gastric cancer is common among older men [26], whereas the diffuse type is reported to be frequent among young women [26]. This discrepancy may occur because men are more likely to drink and smoke than women [26], as confirmed in the current study. In this study, men showed a significantly increased risk of the intestinal type with a higher DII, whereas women presented greater risks of both intestinal and diffuse types with higher DII scores. In general, intestinal type gastric cancer is associated with *H. pylori,* attributable to atrophic gastritis, chronic gastritis, and intestinal metaplasia, and related to well-differentiated and distinctive margins and the formation of tubules or glands [34, 35]. The diffuse type is also associated with *H. pylori* but not with atrophic gastritis and intestinal metaplasia; in addition, the diffuse type is related to less differentiated, infiltrative to neighboring structures, indistinctive margins and family history. [34, 35] The diffuse type is more malignant, with a poorer prognosis than the intestinal type [26], indicating the importance of histological type for the prognosis of gastric cancer [26].

This study has several strengths. First, a previously validated food frequency questionnaire (FFQ) was applied [36]. Second, subgroup analyses characterized histological differences, specifically, intestinal and diffuse types of gastric cancer. These analyses allowed us to identify particular characteristics according to the histological type of gastric cancer. Third, we included information on *H. pylori* infection, a major risk factor for gastric cancer, and we further investigated the associations stratified by *H. pylori* infection. However, this study also has limitations that must be considered when interpreting our results. This case-control study may have been subject to recall bias and selection bias. The cases might have had better recall than the controls, and the participants in the control group might have been more health-conscious than those in the case group. In addition, reverse causality is possible; some of the controls who reported having less pro-inflammatory diets had altered their diets due to subclinical symptoms of disease.

In conclusion, in a case-control study of 388 cases and 776 controls, we observed a significantly positive association between a higher pro-inflammatory diet and a greater risk of gastric cancer, even after adjusting for potential confounding factors. In particular, in the subgroup analysis according to histological type, the risk of intestinal type gastric cancer increased as DII scores increased among men and individuals with *H. pylori* positive infection. Our findings suggest that diet-related inflammation has an important role on gastric cancer risk. Future studies investigating genetic susceptibility to inflammation-related polymorphisms are suggested.

## MATERIALS AND METHODS

### Study population

**Figure 1:**
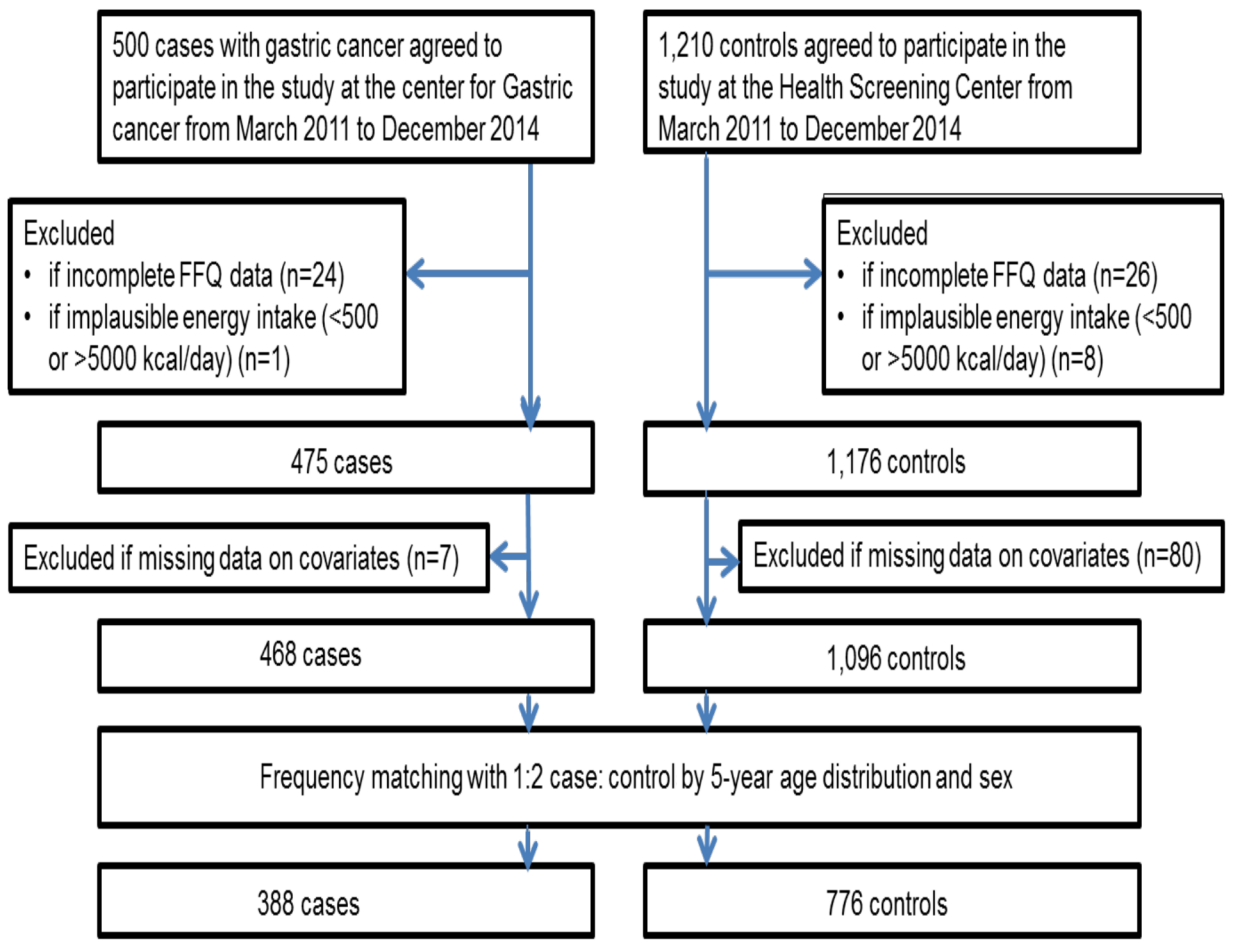
Flow diagram of the study participants

Study participants were enrolled from the Korean National Cancer Center (NCC) from March 2011 to December 2014. The details have been previously described [37]. Briefly, cases (ICD 10 code: C16) were defined as individuals who were newly diagnosed with early gastric cancer within three months and whose diagnoses were histologically confirmed at the NCC. The early stage of gastric cancer was identified as carcinomas restricted to the mucosa or submucosa [38]. Based on Lauren’s histological classification, study participants with non-cardia gastric cancer were categorized into intestinal or diffuse types [39]. Individuals with advanced gastric cancer, another type of cancer, severe mental disease, systematic disease, or diabetes mellitus and women who were pregnant or breast-feeding were excluded. Controls were recruited during the same period from the population of adults receiving health screening examinations at the NCC as part of a benefit program of the National Health Insurance Service. Those with gastric or duodenal ulcers, cancer, diabetes mellitus, or previous treatment for *H. pylori* infection were excluded. At enrollment, 1,710 individuals agreed to participate. Of those, 50 individuals were excluded for providing incomplete dietary data. Of the remaining 1,660 participants, 9 were excluded for reporting an implausible range of total energy intake of <500 kcal or >5000 kcal. Eighty seven adults were additionally excluded from the remaining 1,651 due to missing information regarding BMI (n=9), *H. pylori* infection (n=1), physical activity (n=8), education (n=65), or family history of gastric cancer (n=4). Of the remaining 1,564 participants, 388 cases and 776 controls were finally included based on frequency matching by age (within 5 years) and sex using a 1:2 ratio. As a result, 1,164 participants were included in the final analyses (Figure 1). All participants agreed to voluntarily participate in this study and provided written informed consent. All protocols for the current study were approved by the Institutional Review Board (IRB) of the NCC (IRB Number: NCCNCS-11-438). All methods for the current study were in accordance with the guidelines and regulations of the IRB.

### Dietary assessment and scoring of the DII

A semi-quantitative FFQ including 106 food items was utilized to examine dietary intake. Usual food intake over the previous year was assessed by asking the study participants how frequently they ate each food item, with frequency rated as one of nine categories: never or rarely, once a month, two or three times a month, once or twice a week, three or four times a week, five or six times a week, once a day, twice a day, and three times a day. The validity as well as reproducibility of the FFQ have been previously confirmed and reported [36]. CAN-PRO 4.0 (Computer Aided Nutritional analysis program, Korean Nutrition Society, Seoul, Korea) was used to calculate the daily intake of nutrients for each participant.

To obtain the DII score, dietary assessment data from the FFQ were utilized. The validity of the DII has previously been examined [16]. The methods used to calculate the DII scores followed the protocols outlined by Shivappa and colleagues, who developed the DII [15]. After excluding food components with many missing values, the DII for 35 food components was calculated; these components included vitamin B_12_, vitamin B_6_, β-carotene, carbohydrate, cholesterol, total fat, fiber, folic acid, garlic, ginger, Fe, Mg, monounsaturated fatty acids (MUFAs), niacin, *n-3* fatty acids, *n-6* fatty acids, onion, protein, polyunsaturated fatty acids (PUFAs), riboflavin, saturated fat, Se, thiamin, vitamin A, vitamin C, vitamin D, vitamin E, Zn, green/black tea, flavan-3-ol, flavonols, flavones, flavanones, isoflavones and anthocyanidins. The residual method was used to adjust for total energy intake [40]. As the most frequently used energy adjustment method, this residual method is to estimate the ultimate nutrient effect uncorrelated with total energy intake by utilizing a regression model with total energy intake as an independent variable and the intake of each nutrient as a dependent variable [40].

### Assessment of covariates

All participants were asked to complete self-administered questionnaires that included items on demographics, lifestyle characteristics, and medical history. A positive result on at least one of the following tests was sufficient to confirm *H.pylori* infection: a rapid urease test (Pronto Dry, Medical Instruments Corporation, Solothurn, Switzerland), histology, or serology.

### Statistical analyses

The general characteristics of the study sample were examined using t-tests and chi-square tests. The descriptive characteristics of the study participants were assessed according to the DII tertiles. The normality of the DII was checked. The distribution of the DII tertiles in the control group was as follows: T1: <0.96, T2: 0.96-2.97, and T3: ≥2.97. To investigate the association between DII and the risk of gastric cancer, multivariable logistic regression models were constructed to estimate ORs and 95% CIs. The potential confounding factors were total caloric intake, BMI (kg/m^2^), education, smoking (pack-years), drinking (ethanol amount, in grams per day), physical activity, *H. pylori* infection, and a first-degree family history of gastric cancer. In particular, the covariate variable of smoking was measured in pack-years, which was calculated as the number of packs per day multiplied by the number of years of smoking. The variable of physical activity was measured with the question, ‘Do you regularly exercise? (Yes/No)’. Furthermore, for the subgroup analyses, histological types, i.e., intestinal and diffuse, were examined and additionally stratified by sex and *H. pylori* infection. A two-tailed p-value <0.05 was considered significant. Statistical analyses were performed using SAS version 9.3 (SAS Institute Inc. Cary, NC, USA).
